# COMPARATIVE ANALYSIS OF SURGICAL HEMOSTATIC SPONGES IN LIVER INJURY: STUDY IN RATS

**DOI:** 10.1590/0102-672020180001e1342

**Published:** 2018-03-01

**Authors:** Carlos Edmundo Rodrigues FONTES, Marino Jose MARDEGAM, Orlando Ribeiro PRADO-FILHO, Marcos Victor FERREIRA

**Affiliations:** 1 State University of Maringá, Maringá, PR, Brazil

**Keywords:** Hemostasis, Wounds, Injuries, Liver., Ferimentos, Lesões, Fígado, Hemostáticos.

## Abstract

**Background:**

Obtaining effective hemostasis either in the traumatic or surgical lesions of parenchymal viscera, especially the liver, has always been a challenge.

**Aim:**

Comparative study between the use of different hemostatic sponges in hepatic wound and their capacity of integration to cells in a short period.

**Methods:**

Fifteen Wistar rats were divided into three groups. Through laparotomy a standardized wound in hepatic right lobe was made. The animals were treated with three sponges, being gelatin in group I, equine collagen in group II, and oxidized cellulose in group III. The hemostatic capacity was analysed. On the 7º day after surgery samples for histology analysis (H&E and picrosirius) were collected for inﬂammatory evaluation and collagen quantification (types I and III) with polarized microscopy.

**Results:**

All materials used had similar haemostatic effects, with no significant difference in hemostasis time. In the assessment of tissue repair and adhesions provoked, as well as analysis of the inflammatory process, the gelatin sponge presented greater inflammation and adhesions to the contiguous structures to the procedure in relation to the other groups.

**Conclusion:**

Animals which had their wounds treated with collagen and regenerated cellulose sponges presented better results in relationship to the ones treated with gelatin sponge.

## RESUMO

**Racional:**

A obtenção de hemostasia eﬁcaz nas lesões traumáticas ou cirúrgicas de vísceras parenquimatosas, em especial do fígado, sempre foi desafiante.

**Objetivo:**

Comparar o uso de hemostáticos absorvíveis em ferimento hepático quanto à capacidade hemostática e de integração aos tecidos em curto prazo.

**Métodos:**

Foram utilizados 15 ratos Wistar separados em três grupos. Foi realizada laparotomia e ferimento padronizado em lobo hepático direito. Os animais do grupo I foram tratados com esponja de gelatina sobre os ferimentos; os do grupo II com esponja de colágeno equino, e os do grupo III com celulose regenerada oxidada. Na ocasião foi estudada a capacidade hemostática. No 7º dia de pós-operatório nova laparotomia foi realizada e foram coletadas amostras para estudos histológicos (H&E e picrosirius) avaliando os processos por microscopia ótica e de polarização para quantiﬁcação de colágeno (tipos I e III).

**Resultados:**

Todos os materiais usados apresentaram efeitos hemostáticos semelhantes, não havendo diferença signiﬁcativa no tempo de hemostasia. Na avaliação da reparação tecidual e aderências provocadas, assim como análise do processo inﬂamatório, os tratados com esponja de gelatina apresentaram maior inflamação e aderências às estruturas contíguas ao procedimento em relação aos outros grupos.

**Conclusão:**

Os animais tratados com a esponja de colágeno e celulose regenerada apresentaram resultados melhores que aqueles com esponja de gelatina.

## INTRODUCTION

Subsequent hemorrhages of wounds have always caused the search for high power hemostatic agents. These devices are the main goals of surgeons in trials to improve results. To obtain effective hemostasis from traumatic or surgical lesions of parenchymal viscera, especially the liver, has always been a challenge^11^ ^,^ ^12^ ^,^ ^15^. The possibility of use of different substances leaded to the production of collagen and lower inflammatory reaction, when compared with conventional sutures ^2^ ^,^ ^4^ ^,^ ^5^ ^,^ ^10^

In the search for hemostasis of hepatic lesions, in addition to adhesives and materials already known, other studies are available, using gel-filled electrocautery^10^ and fibrin sealants, which have different degrees of adhesion and abscess formation^3^.

In a recent study, biological adhesives of collagen, fibrinogen and thrombin were used in experimental wounds in rats demonstrating their efficacy in the treatment of these lesions, promoting good hemostasis and a low incidence of adherence to neighboring structures^11^.

The present study aimed to study comparatively the use of three surgical sponges as hemostatic agents in hepatic injuries, in order to demonstrate hemostatic and tissue integration capabilities in the short term.

## METHODS

This study was approved by the Research Ethics Committee of the State University of Maringá under number 011/2010COPEP/UEM. Fifteen Wistar rats from the University’s vivarium were used; the average weight was 200 g and distributed in three groups. All the animals had ventral region opened and a standardized 2x2 cm hepatic right lobe wound was produced. Wounds were treated by enveloping them with sponges, being of gelatin in group I; of equine collagen in group II; and of regenerated cellulose oxidized in group III. The hemostatic capacity was analyzed from the observation of the bleeding time of the lesion. On the 7^th^ postoperative day samples were collected for histological studies (H&E and picrosirius), evaluating inflammatory processes (qualitative analysis of the presence of neutrophils, giant cells, granuloma and neovascularization) and, by polarization microscopy, the quantification of collagen (types I and III). The images were captured by AxioCam (Zeiss, Jena, Germany) high resolution camera coupled to the Axioskop Plus (Zeiss) light microscope equipped with 40X objective fluorescence filters (FITC). The images were later analyzed using Image-Pro Plus, version 4.5.029 (Media Cybernetics, Silver Spring, MD, USA).

### Statistical analysis

Results were submitted to Tukey’s test using GraphPad Prism 5.04 software. The level of significance was 5% (p<0.05).

## RESULTS

**FIGURE 1:**
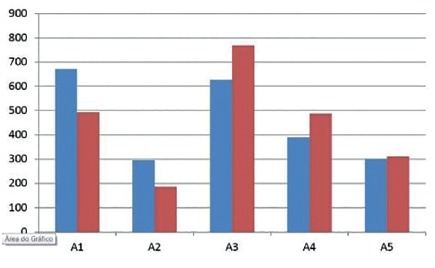
Average and median qualitative of inflammatory process in group 1

**FIGURE 2:**
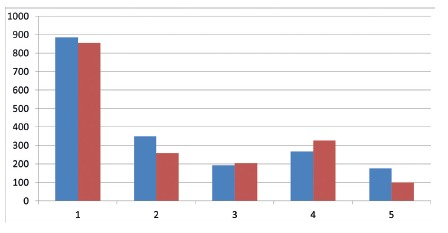
Average and median qualitative of inflammatory process in group 2

**FIGURE 3:**
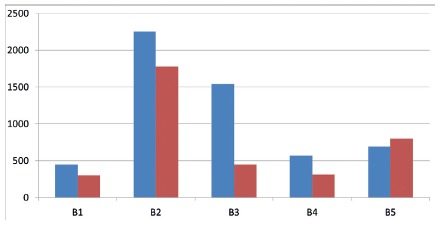
Average and median qualitative of inflammatory process in group 3

All materials used showed similar hemostatic effects, with no significant difference in hemostasis time. On the 7^th^ postoperative day the animals were submitted to a new operative procedure to evaluate the tissue repair and adhesions provoked, as well as the inflammatory process analysis. Sponge-treated gelatin presented greater inflammation and adherence to the structures contiguous to the procedure in relation to the other groups (Figures 1, 2 and 3).

There was no qualitative significance in the histological analysis for the results in the predetermined parameters for inflammatory process and qualitative criteria for the presence of neutrophils, giant cells, granuloma, neovascularization in the three groups. In relation to the quantification of types I and III collagen, however, the segments treated with equine collagen and cellulose showed a greater stimulation of tissue repair, a greater amount of type I collagen was observed. 

**FIGURE 4:**
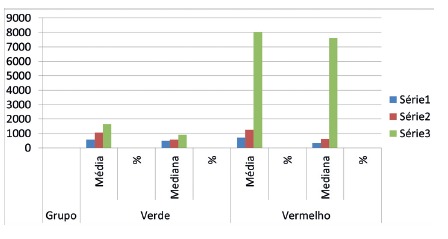
Quantification of collagen type I (red) and type III (green) in the three groups analyzed (p<0.05)

**FIGURE 5:**
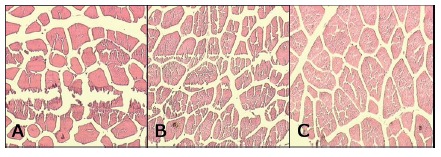
H&E in groups 1 (A), 2 (B) and 3 (C) on the 7th postoperative day (100×)

**FIGURE 6:**
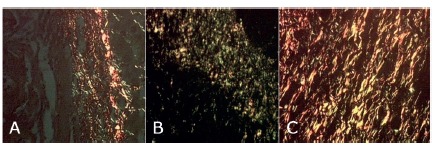
- Collagen type I (red) and type III (green) of the groups 1 (A), 2 (B) and 3 (C) on the 7th postoperative day (polarization microscopy- Picrosirius )

## DISCUSSION

Wistar rats were chosen for this study due to biological characteristics and metabolism increased in relation to men allowing late analysis in relation to the healing process and in lesser time of observation. Another important reason for the choice was the greater ease in obtaining a homogenous sample^6^. The texture and characteristics of the manipulation of the hepatic parenchyma of the animal simulate with great resemblance human liver. Experimentally caused wounds in rat liver have been used by several authors for the study of hemostasis and tissue regeneration over years, and various physical and chemical agents^1^ ^,^ ^9^ ^,^ ^11^ have been used. These studies demonstrated that its use in iatrogenic or traumatic hepatic lesions is feasible, reducing bleeding time and accelerating the process of tissue regeneration. The standardized wound was established from the study of a pilot group. The area and depth chosen were those in which hemostatic procedure would be required. Hemostasis of the injured wound was achieved in the three groups before cavity closure, which occurred in about 3 min after sponge application. Analysis of hemostasis results in all three groups was produced at similar times, with no advantage of one over the other under this criterion.

 The technical part of the application of the sponges did not present difficulties in its use in any group, all being easy to handle.

Healing with adequate tissue regeneration involves a series of biological events in the local of inflammatory response with the formation of connective tissue to reshape the affected area. Taking in consideration all healing phases, it is important to emphasize the fibroplasia that appears after 48 h by invasion of fibroblasts that multiply producing amorphous fundamental substance which will guide the collagen fibers that are responsible for the strength and integrity of the tissue repair.

The scope of this study was to evaluate the use of surgical sponges and their ability to stimulate tissue repair, the appearance of collagen in the wound to be repaired was stimulated. The use of equine collagen sponges, oxidized regenerated cellulose and gelatin creates a decisive doubt during the attempt of hemostasis of which is better. Surgical patches are a good option for such situations, and have already demonstrated good efficacy in several studies^4,5,6,7,9,10,11,13,14^ ; however, they are not always available in hospitals. The sponges in turn are easier to handle and are found in most hospitals. 

This study aimed to respond to the clinical doubt in the use of an already widely used material - oxidized regenerated cellulose sponge - and the gelatine sponge.

The qualitative analysis using conventional microscopy and H&E staining of the resected hepatic segments did not show statistically significant differences in the inflammatory process. 

When the three groups were submitted to statistical analysis, it was evident that the equine collagen sponge and the regenerated cellulose sponge stimulated the appearance of collagen in tissue repair more than gelatin did. (Figure 4).

Taking in consideration obtained data, it can be stated that from the point of view of hemostasis and the inflammatory process there is no significant difference among the three groups. However, the equine collagen and regenerated cellulose sponges contributed more than that of gelatin, with the appearance of a greater amount of collagen types I and III, which provides a more favorable repair process (Figures 5 and 6).

## CONCLUSION

The sponges of collagen and regenerated cellulose presented higher amounts of collagen in the injured area, demonstrating a greater stimulus in tissue repair in comparison the gelatin sponge. The regenerated cellulose sponge stimulated the production of type I collagen more than that of collagen did. There were no significant statistical differences either in the hemostatic or in the inflammatory aspects among the three groups.
